# Physiological and Biochemical Responses of *Nostoc linckia* to Metal Oxide Nanoparticles

**DOI:** 10.3390/life15091477

**Published:** 2025-09-20

**Authors:** Liliana Cepoi, Vera Potopová, Ludmila Rudi, Tatiana Chiriac, Svetlana Codreanu, Ana Valuta, Valeriu Rudic

**Affiliations:** 1 Institute of Microbiology and Biotechnology, Technical University of Moldova, MD 2028 Chisinau, Moldova; ludmila.rudi@imb.utm.md (L.R.); tatiana.chiriac@imb.utm.md (T.C.); svetlana.codreanu@imb.utm.md (S.C.); ana.valuta@imb.utm.md (A.V.); valeriu.rudic@imb.utm.md (V.R.); 2 Faculty of Agrobiology, Food and Natural Resources, Department of Agroecology and Crop Production, Czech University of Life Sciences Prague, Kamýcká 129, 165 00 Praha-Suchdol, Czech Republic; potop@af.czu.cz

**Keywords:** ZnONPs, TiO_2_NPs, *Nostoc linckia*, biomass composition, pigments, stress conditions

## Abstract

Metal oxide nanoparticles, such as ZnONPs and TiO_2_NPs, are increasingly applied in various industries. However, their effects on photosynthetic microorganisms at environmentally relevant concentrations remain poorly understood. This study evaluated the impact of ZnONPs and TiO_2_NPs, at concentrations ranging from 0.1 to 30 mg/L, on the cyanobacterium *Nostoc linckia* (strain CNMN-CB-03), a species recognized for its adaptability and biotechnological potential. The nanoparticles were added to controlled cultures, and changes in biomass composition and pigment content were assessed using spectrophotometric assays. Both nanoparticle types significantly affected the physiological and biochemical profile of *Nostoc linckia*. Low concentrations of ZnONPs stimulated the accumulation of biomass, chlorophyll, carotenoids, and lipids, while higher doses caused a reduction in phycocyanin and in total phycobiliproteins content. TiO_2_NPs consistently promoted biomass growth across all tested concentrations, with decrease in carotenoids and total phycobiliproteins observed at the highest concentrations. For both nanoparticle types, malondialdehyde (MDA) levels decreased compared to the control, indicating reduced oxidative stress and effective cellular adaptation. The results highlight the remarkable resilience and metabolic flexibility of *Nostoc linckia* in the presence of nanoparticles, supporting its potential as a biotechnological platform for the sustainable production of valuable metabolites under controlled stress conditions.

## 1. Introduction

Metallic nanoparticles, such as zinc oxide (ZnONPs) and titanium dioxide (TiO NPs), are among the most extensively studied nanomaterials due to their broad functional versatility and high application potential in various fields, including environmental remediation, water purification, medicine, cosmetics, and agriculture [1]. These nanomaterials exhibit common physicochemical features, including photocatalytic activity, the capacity to generate reactive oxygen species (ROS), chemical stability, and nanoscale dimensions, all of which enable strong interactions with biological systems [2,3]. Nevertheless, their performance in biological environments raises concerns regarding potential toxicological as well as adaptive responses in photosynthetic organisms, particularly within aquatic ecosystems, and, indirectly, their implications for human health [1,4,5].

The effects of ZnONPs and TiO_2_NPs on microalgae and cyanobacteria are influenced by their chemical composition, particle shape and size, exposure concentration, and the biological characteristics of the species. Although numerous studies report toxic or neutral effects, there are cases where nanoparticles can induce stimulatory effects. This outcome depends not only on concentration and species, but also on the synthesis route and the mode of exposure [2].

Most publications demonstrating the toxicity of ZnONPs and TiO_2_NPs are based on studies employing high concentrations. Indeed, concentrations of 200–400 mg/L ZnNPs and TiO_2_NPs added to microalgal culture media have been shown to induce severe oxidative stress, resulting in decreased biomass, damage to the photosynthetic apparatus, and disruption of membrane systems [6,7]. These effects are common to both nanoparticle types and do not significantly depend on the microalgal species. At lower concentrations, the observed effects depend on the type of nanoparticles and the biological traits of the photosynthetic microorganisms [8,9,10]. For both types of nanoparticles, deterioration of the photosynthetic systems has been reported, a mechanism attributed to particle aggregation or ion dissolution [11,12,13,14].

Few studies report stimulatory effects on growth and biomolecule accumulation resulting from the action of ZnONPs and TiO_2_NPs [9,15,16]. The results of these studies highlight that ZnONPs and TiO_2_NPs can exert stimulatory effects under certain conditions, which are influenced by the species involved, the applied dose, and the mode of exposure. In this context, there is a clear need to move beyond simple toxicity assessments based on high concentrations and to conduct research focused on the effects of these nanomaterials on specifically identified photosynthetic organisms, strategically selected as biotechnological targets. Such an approach would enable the controlled exploitation of their potential in biotechnology and contribute to defining application limits tailored to each biological system.

Interest in Cyanobacteria of the genus *Nostoc* remains high, as several species exhibit significant potential for biotechnological applications due to their remarkable adaptability to changes in cultivation conditions. Species from this genus are extensively studied as producers of exopolysaccharides and phycobiliproteins [17,18]. Applied studies also reported their use in bioremediation [19,20]. In most cases, technologies developed to enhance the synthesis of bioactive compounds are based on modifying the cultivation regime and the controlled induction of oxidative stress, with varying degrees of intensity, as a strategy for metabolic stimulation [21,22].

With climate change and drought increasingly threatening food security, the stress resilience of photosynthetic microorganisms such as *Nostoc linckia* offers valuable potential for sustainable biotechnological applications [23]. In this context, *Nostoc linckia* has been used in green synthesis of nanoparticles and has also become an important research subject in studies investigating the impact of nanoparticles on photosynthetic organisms [24,25,26].

This study aims to evaluate the effects of zinc oxide and titanium dioxide nanoparticles on the cyanobacterium *Nostoc linckia*, considered a biotechnologically relevant organism, with a focus on the metabolic responses associated with both toxic and stimulatory effects. The objective is to identify the optimal conditions for the controlled application of these nanomaterials in nanobiotechnological processes.

## 2. Materials and Methods

### 2.1. Nanoparticles Used

In this study, the following types of nanoparticles were used: zinc oxide nanoparticles (ZnONPs; product code 721077, Sigma-Aldrich, Merck KGaA, Darmstadt, Germany), with a particle size of <100 nm (TEM); and titanium dioxide nanoparticles (TiO_2_NPs; product code 718467, Sigma-Aldrich, Merck KGaA, Darmstadt, Germany), with a particle size of 21 nm (TEM) and a purity of ≥99.5%. To disperse the TiO_2_NPs and prevent aggregation, the nanoparticle suspensions in deionized water were sonicated at a frequency of 22 kHz and an ultrasonic intensity of 7 W/cm^2^ for 5 min in an ice bath.

### 2.2. Cyanobacterial Strain and Cultivation Conditions

In this study, the *Nostoc linckia* (Roth) Born et Flah, strain CNMN-CB-03, was used. The strain is maintained in the National Collection of Non-Pathogenic Microorganisms at the Institute of Microbiology and Biotechnology, Technical University of Moldova. For the experiments, a mineral nutrient medium with the following composition was used: KNO_3_—0.5 g/L; K_2_HPO_4_—0.45 g/L; NaHCO_3_—0.05 g/L; MgSO_4_·7H_2_O—0.1 g/L; CaCl_2_—0.11 g/L; ZnSO_4_·7H_2_O—0.00005 g/L; MnSO_4_—0.002 g/L; H_3_BO_3_—0.00085 g/L; (NH_4_)_6_Mo_7_O_24_·4H_2_O—0.00225 g/L; FeSO_4_·7H_2_O—0.004 g/L; Co(NO_3_)_2_·H_2_O—0.000009 g/L; and EDTA—0.00475 g/L. Cultivation was carried out in 100 mL Erlenmeyer flasks containing 50 mL of suspension, under controlled laboratory conditions: medium pH between 6.8 and 7.2, constant temperature of 25–27 °C, continuous illumination at an intensity of 55 μmol photons/m^2^/s, and periodic slow agitation. The inoculum culture was obtained under the same conditions and applied at a concentration of 0.4 g/L dry weight. ZnONPs and TiO_2_NPs were added at 0.1, 1.0, 10.0, 20.0, and 30.0 mg/L, and the cultivation lasted for 12 days.

### 2.3. Determination of Biomass Quantity and Preparation of Samples

Biomass content was determined spectrophotometrically at 590 nm, using a calibration curve correlating absorbance with dry biomass (g/L). The calibration curve was established from multiple replicates by measuring the absorbance of culture samples, followed by centrifugation (5000× *g*, 10 min), washing twice with 2% ammonium acetate to remove salts, and drying the pellets to constant weight. The resulting linear relationship between optical density and dry biomass was then used to calculate the biomass concentration in experimental samples.

For subsequent biochemical analyses, a standardized biomass suspension was prepared. After centrifugation and washing as described above, biomass was resuspended in distilled water to obtain a final concentration of 10 mg/mL. This standardized suspension was subjected to six consecutive freeze–thaw cycles to disrupt the cells and was then stored at −20 °C. The standardized material was used as a common reference for all biochemical assays, ensuring comparability of results across experiments.

### 2.4. Determination of Protein Content in Biomass

Protein content was determined using the Lowry method [27]. Proteins were extracted by treating 0.1 mg of biomass with 0.9 mL of 0.1 N NaOH solution for 30 min. The mixtures were incubated at room temperature for 30 min. A 0.2 mL aliquot was diluted with 0.8 mL distilled water, then mixed with 1.5 mL of a complex reagent (49 mL of 2% Na_2_CO_3_ in 0.1 N NaOH and 1 mL of 0.5% CuSO_4_ in 1% sodium potassium tartrate). After 10 min, 0.5 mL of Folin–Ciocalteu reagent (1:3 dilution of water) was added. Absorbance was measured at 750 nm after 30 min, and protein content in mg/mL in the assay extract was calculated using a bovine serum albumin calibration curve. The results were then recalculated as % of dry weight, using the dry biomass determined for each sample.

### 2.5. Determination of Carbohydrate Content in Biomass

Carbohydrate content was measured spectrophotometrically with the Anthrone reagent in acidic medium [28]. For each sample, 0.2 mL biomass suspension was mixed with 2 mL of 0.5% Anthrone reagent in concentrated sulfuric acid, heated for 10 min in a water bath, cooled, incubated for 10 min at room temperature, and the absorbance was measured at 620 nm. Carbohydrate content was calculated using a glucose calibration curve in mg/mL in the assay extract. The results were then recalculated as % of dry weight, using the dry biomass determined for each sample.

### 2.6. Determination of Chlorophyll a and Carotenoid Content

Photosynthetic pigments were extracted in 96% ethanol (10 mg biomass in 1 mL, dark, 12 h, room temperature), centrifuged, and absorbance was measured at 450 nm (carotenoids), 649 nm, and 665 nm (for chlorophyll a). Concentrations were calculated according to [29] and expressed as a % of dry biomass.

### 2.7. Determination of Phycobiliprotein Content

Phycobiliproteins were determined spectrophotometrically in aqueous extracts obtained by centrifugation of standardized biomass samples. Absorbance was measured at 565 nm for phycoerythrin (PE), 620 nm for phycocyanin (PC), and 650 nm for allophycocyanin (APC). Phycobiliprotein content was calculated according to [30] and expressed as a % of dry biomass.

### 2.8. Determination of Lipid Content

Lipid content was measured using the phospho-vanillin colorimetric assay [31]. For lipid extraction, 10 mg of biomass was mixed with 1 mL of a chloroform: ethanol mixture (9:1, *v*/*v*) and stirred continuously at room temperature for 120 min. The resulting lipid extract was hydrolyzed with 66% sulfuric acid, and 0.1 mL of the hydrolysate was combined with 2.9 mL of phospho-vanillin reagent. After 30 min of color development, absorbance was read at 560 nm. Lipid concentration was calculated using a calibration curve prepared with oleic acid and expressed as % of dry biomass.

### 2.9. Determination of Malondialdehyde (MDA) Content

Malondialdehyde (MDA) in biomass was quantified using the thiobarbituric acid (TBA) reactive substances assay [32]. For each sample, 10 mg of biomass was mixed with 3.0 mL of 0.76% TBA dissolved in 20% trichloroacetic acid. The reaction mixtures were incubated at 95 °C for 20 min, and then rapidly cooled. Absorbance was measured at 532 nm and 600 nm to correct for nonspecific turbidity. MDA content was calculated using the specific molar extinction coefficient specific to the MDA–TBA complex.

### 2.10. Statistical Analysis

Experiments were conducted in triplicate, and results are reported as mean ± standard deviation (SD). Statistical evaluation focused primarily on comparisons between each treatment and the control. One-way ANOVA was applied separately for each parameter (biomass, proteins, carbohydrates, pigments, lipids, MDA) under ZnO and TiO_2_ NP treatments to assess overall differences among concentrations. Homogeneity of variances was examined empirically by comparing group variances. When variances were of similar magnitude, post hoc pairwise comparisons used two-sample tests assuming equal variances; when heteroscedasticity was evident, post hoc pairwise comparisons used two-sample tests assuming unequal variances. Statistical significance was set at *p* ≤ 0.05.

## 3. Results

### 3.1. Biomass Accumulation

**Figure 1:**
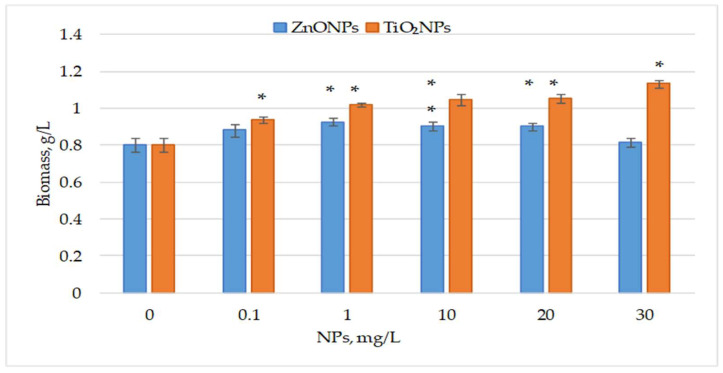
Biomass (g/L, dry weight) of the cyanobacterium *Nostoc linckia* after 12 days of cultivation in the presence of ZnO and TiO_2_ nanoparticles at different concentrations (mg/L); 0—control. Data are presented as mean ± SD (n = 3). Statistical significance is indicated relative to the control: *—*p* < 0.05.

Figure 1 illustrates the effect of ZnO and TiO_2_ nanoparticles, applied from day 1 of cultivation, on the final biomass of the cyanobacterium *Nostoc linckia*.

ZnO nanoparticles increased final biomass by 9.51% and 15.11% (*p* < 0.05) at 0.1 and 1 mg/L, respectively. At 10 and 20 mg/L, biomass increased by over 12% (*p* < 0.05), while the highest concentration had no significant effect. In contrast, all tested concentrations of TiO_2_ nanoparticles significantly enhanced biomass, ranging from 16.98% (*p* < 0.05) at 0.1 mg/L to 41.04% (*p* < 0.05) at 30 mg/L. Concentrations between 1 and 20 mg/L yielded highly significant increases of 26.49% to 31.15%. ANOVA showed overall differences among concentrations of ZnO NPs, F(5, 12) = 11.90, *p* = 0.00026; and among concentrations of TiO_2_ NPs, F(5, 12) = 58.68, *p* = 5.13 × 10^−8^ (S1), indicating that concentration of this type of nanoparticle is a factor influencing the amount of biomass produced. However, given the limited number of replicates (n = 3), this conclusion should be interpreted with caution.

### 3.2. Protein and Carbohydrate Content

**Figure 2:**
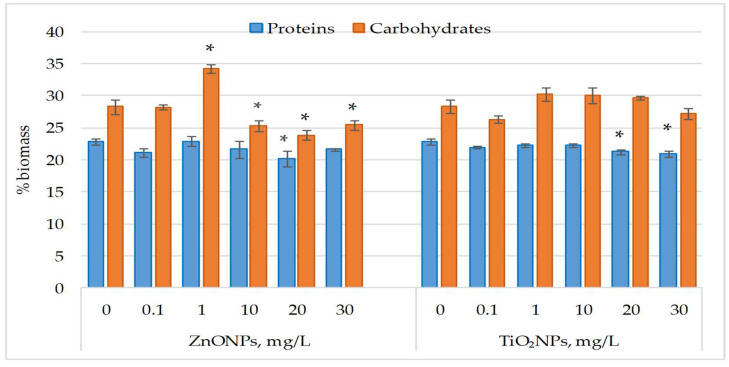
Protein and carbohydrate content (% in dry biomass) in *Nostoc linckia* biomass following exposure to ZnO and TiO_2_ nanoparticles at different concentrations (mg/L); 0—control; values represent mean ± SD (n = 3). Statistical significance is indicated relative to the control: *—*p* < 0.05.

Figure 2 displays the changes in protein and carbohydrate content in *Nostoc linckia* biomass after exposure to ZnO NPs and TiO_2_ NPs.

ZnO nanoparticles had no significant effect on protein content in *Nostoc linckia* biomass at most tested concentrations, except at 20 mg/L, which caused an 11.60% reduction (*p* < 0.05). Similarly, TiO_2_NPs at 30 mg/L significantly decreased protein content by 8.45% (*p* < 0.05). ZnONPs at 1 mg/L induced a 21.15% increase in carbohydrate content (*p* < 0.05), whereas concentrations of 10–20 mg/L caused decreases of 9.80% (*p* < 0.05) to 15.54% (*p* < 0.05). Carbohydrate content changes induced by TiO_2_NPs were not significant, varying from 5.09% to 6.81% compared to the control.

### 3.3. Chlorophyll and Carotenoid Content

**Figure 3:**
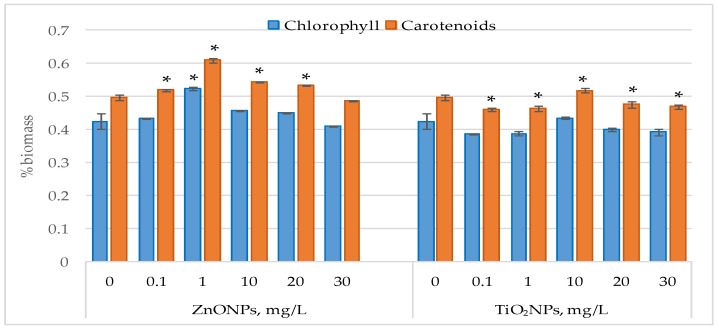
Chlorophyll and carotenoid content (% in dray biomass) in *Nostoc linckia* biomass following exposure to ZnO and TiO_2_ nanoparticles at different concentrations (mg/L); 0—control; values represent mean ± SD (n = 3). Statistical significance is indicated relative to the control: *—*p* < 0.05.

Figure 3 illustrates the changes in photosynthetic pigment content in *Nostoc linckia* biomass following exposure to ZnO nanoparticles and TiO_2_ nanoparticles.

Exposure of *Nostoc linckia* cultures to ZnO nanoparticles did not cause significant modification in chlorophyll content, except the concentration of 1 mg/L which led to a 23.0% increase in chlorophyll content (*p* < 0.05), All tested concentrations of ZnONPs also led to increases in carotenoid content in the biomass of *Nostoc linckia*. At 1 mg/L, carotenoid content increased by 22.76% (*p* < 0.05), while increases of 9.29% (*p* < 0.05) and 7.42% were recorded at 10 and 20 mg/L, respectively. Supplementation of the growth medium with TiO_2_ nanoparticles at concentrations of 0.1 and 1 mg/L resulted in reduction in carotenoid content, by 7.24%) and 6.56% (*p* < 0.05). At concentrations of 20 and 30 mg/L, no modifications in chlorophyll content were observed, while carotenoids decreased by 4.09% (*p* < 0.05) and 5.54% (*p* < 0.05), respectively. At a concentration of 10 mg/L of zinc oxide nanoparticles, the amount of carotene in nostoc biomass increased

### 3.4. Phycobiliproteins Leve

**Figure 4:**
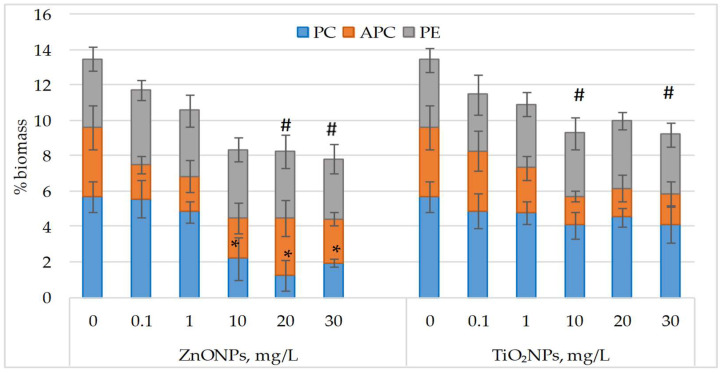
Phycobiliprotein content (phycocyanin (PC), allophycocyanin (APC), phycoerythrin (PE); % of dry biomass) in *Nostoc linckia* biomass following exposure to ZnO and TiO_2_ nanoparticles at different concentrations (mg/L). 0—control; data are presented as mean ± SD (n = 3). Statistical significance is indicated relative to the control: *—*p* < 0.05 for individual phycobiliproteins; #—*p* < 0.05 for sum of phycobiliproteins.

Exposure to ZnO and TiO_2_ nanoparticles reduced the content of accessory photosynthetic pigments in *Nostoc linckia*, with the corresponding phycobiliprotein levels shown in Figure 4.

Analysis of the obtained data shows that the individual contents of the phycobiliproteins, when considered separately, did not differ significantly from the control in most experimental variants. The only exceptions were observed in the treatments with zinc oxide nanoparticles at concentrations of 10, 20, and 30 mg/L, where *Nostoc linckia* biomass contained 61.5–77.9% less phycocyanin compared to the control. In the other cases, although a general decreasing trend in phycobiliproteins was noticeable, the differences were not statistically significant. However, when the sum of phycobiliproteins in *Nostoc linckia* biomass was calculated, a significant reduction in this indicator was recorded at the higher concentrations of both types of nanoparticles.

### 3.5. Lipid Content and Malondialdehyde (MDA) Levels

**Figure 5:**
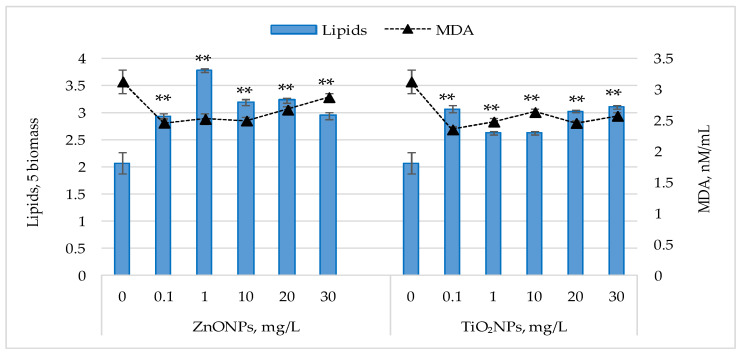
Lipid content (% in dry biomass) and malondialdehyde MDA levels (nM/mL) in *Nostoc linckia* biomass following exposure to ZnO and TiO_2_ nanoparticles at different concentrations (mg/L). 0—control; data are presented as mean ± SD (n = 3). Statistical significance is indicated relative to the control: **—*p* < 0.05.

Figure 5 shows the changes in lipid content and malondialdehyde (MDA) levels following exposure of *Nostoc linckia* cultures to ZnONPs and TiO_2_NPs.

For both types of nanoparticles, an increase in lipid content was observed in *Nostoc linckia* biomass at all tested concentrations. ZnONPs induced significant increases (*p* < 0.05), ranging from 42.02% to 82.35%, with the most pronounced effect observed at 1 mg/L. Concentrations of 0.1 and 30 mg/L resulted in similar increases (~42%), while 20 and 30 mg/L led to increases of 53.95–56.30%. For TiO_2_NPs, lipid content increased by 26.89% (*p* < 0.05) to 50.25% (*p* < 0.05). The highest values were observed at 0.1 mg/L (46.39%), 20 mg/L (48.40%), and 30 mg/L (50.25%), whereas 1 and 10 mg/L caused moderate increases of 26.89% (*p* < 0.05)—27.06% (*p* < 0.05).

Malondialdehyde (MDA) levels in the harvested biomass were significantly lower (*p* < 0.001) than in the control. Exposure of *Nostoc linckia* culture to ZnONPs at 0.1–10 mg/L resulted in MDA reductions of 19.23–21.25%, while 20 and 30 mg/L caused decreases of 14.51% and 8.06% (*p* < 0.01), respectively. For TiO_2_NPs, MDA levels were 15.75% (*p* < 0.001)—24.64% (*p* < 0.001) below control, with the greatest reductions at 0.1 mg/L, similar to the results obtained for ZnONPs. The results shown in Figure 5 indicate an inverse relationship between lipid content and MDA levels in *Nostoc linckia*. Statistical analysis confirmed this negative correlation, with correlation coefficients of −0.78 for ZnONPs and –0.88 for TiO_2_NPs.

For both parameters, the variance among experimental groups was small. The results of one-way ANOVA showed overall differences among concentrations of ZnO NPs, F(5, 12) = 112.02, *p* = 1.22 × 10^−9^ and of TiO_2_NPs, F(5,12) = 63.79, *p* = 3.1 × 10^−8^ for lipid content. ANOVA also showed overall differences among concentrations of ZnO NPs, F(5,12) = 43.49, *p* = 2.8 × 10^−7^ and of TiO_2_NPs, F(5,12) = 60.50, *p* = 4.3 × 10^−8^ for MDA level (S1). These findings suggest that nanoparticle concentration influences both lipid accumulation and MDA levels in *Nostoc linckia*, although the interpretation is limited by the low number of replicates (n = 3).

Overall, exposure of *Nostoc linckia* cultures to ZnO and TiO_2_ nanoparticles significantly affected biomass accumulation, biochemical composition, and photosynthetic pigments.

## 4. Discussion

In the present study, zinc oxide and titanium dioxide nanoparticles with photocatalytic activity were applied at concentrations ranging from 0.1 to 30.0 mg/L. According to the literature, these nanoparticles have previously been tested across a wide concentration range (0.1 up to 400–500 mg/L), and the observed effects depend on both the type of nanoparticles and the exposed microorganism, being primarily concentration dependent. At higher concentrations, the response of microorganisms is typically associated with severe oxidative stress [33].

In this study, the model organism selected was the cyanobacterium *Nostoc linckia*, a species characterized by a wide diversity of natural habitats and a high capacity to adapt to changing environmental conditions [20]. This adaptability makes it suitable for investigating responses to nanoparticle exposure and for evaluating the mechanisms of tolerance and adaptation to stress factors.

The results obtained showed that both types of nanoparticles, at certain concentrations within the tested range, stimulated biomass growth in *Nostoc linckia*. This effect was more pronounced for TiO_2_NPs and varied slightly depending on concentration, showing a tendency to increase as nanoparticle levels rose. In contrast, for ZnONPs, the positive effect on biomass was greater at lower concentrations and declined at higher levels, indicating the presence of a specific tolerance threshold. These differences may reflect the intrinsic solubility of the two nanomaterials: TiO_2_NPs are largely insoluble under culture conditions, while ZnONPs can release Zn^2+^ ions in measurable amounts. Since the culture medium contains a small amount of EDTA, added solely to stabilize iron and other micronutrients, its influence on metal speciation cannot be completely ignored. Nevertheless, given the very low concentration applied, EDTA is unlikely to have significantly altered the dissolution behavior of ZnONPs and their effects on *Nostoc linckia.* These findings highlight both the ability of the culture to adapt to ZnONPs and TiO_2_NPs in the growth medium and the potential to exploit the interaction between cyanobacteria-nanoparticle interactions to stimulate biomass production. Biomass increase indicates the absence of obvious toxicity, although the possibility of underlying metabolic stress cannot be excluded.

Stimulation of biomass production in response to nanoparticle exposure is rarely reported in the literature; most studies describe inhibitory or relatively neutral effects. For example, the cyanobacterium *Limnospira platensis* (formerly *Srthrospira platensis*) (Cyanophyta) responded to interaction with TiO_2_NPs by reducing biomass at low concentrations, with no significant changes at higher concentrations. In contrast, exposure to ZnONPs (with the size < 100 nm) led to a significant decrease in biomass [34]. The microalga *Tetraselmis suecica* (Chlorophyta) exhibited a 40% reduction in biomass after 72 h of exposure to TiO_2_NPs (with the size 10–50 nm) at a concentration of 7.9 mg/L [8]. Similarly, *Nannochloropsis oculata* showed biomass decreases after 72 h of exposure to ZnONPs (size 10–30 nm) above 10mg/L. Prolonged exposure (96 h) to ZnONPs led to a 15–40% reduction in biomass at concentrations of 10–50 mg/L, whereas lower concentrations had no significant effect on biomass content [9].

There are also examples of stimulatory effects, such as those observed with TiO_2_NPs with size of 25 nm added to mineral growth media at 50–300 mg/L, where a significant increase in biomass accumulation was found in the microalgae *Scenedesmus quadricauda* (Chlolorophyta) and *Stigeoclonium tenue* (Chlolorophyta), with a clear dependence on species and concentration [35]. The microalga *Haematococcus lacustris* responded to interaction with TiO_2_ (size 21 nm) nanoparticles with a significant biomass increase during the green stage, an effect that was also concentration dependent. Zinc oxide nanoparticles (<100 nm) likewise showed specific concentrations at which biomass stimulation occurred [34]. The cyanobacterium *Nostoc linckia*, exposed to CuONPs, (50 nm) exhibited a 13.6–24.6% increase in biomass at 10–30 mg/L [26]. These results appear to be exceptions and are most likely attributable to hormesis effect [33].

Analyzing the obtained results, ZnONPs were found to stimulate biomass accumulation at low concentrations, whereas TiO_2_NPs induced a concentration-dependent increase in biomass across the tested range. These observations indicate that the response of the cyanobacterium *Nostoc linckia* depends not only on concentrations but also on nanoparticle type, suggesting the involvement of specific interaction and adaptation mechanisms.

Another important parameter in analyzing the effects of nanoparticles on microalgae and Cyanobacteria is the protein content. An integrated assessment of both biomass accumulation and protein levels is important for determining the nature of the processes occurring in the culture. It is unlikely that biomass increase reflects hormetic effects when protein content remains at the control levels. Thus, in experimental variants, protein content was not significantly altered, and biomass accumulation cannot be attributed to hormesis. The relationship between biomass and protein content varies among cyanobacteria and microalgae exposed to TiO_2_NPs and ZnONPs. For example, exposure of *Nostoc linckia* to CuONPs at 10 and 20 mg/L increased biomass, without altering protein content, suggesting that the photoactive nanoparticles did not induce protein oxidation processes and that biosynthetic processes remained almost unaffected [26]. In contrast, other species showed concentration-dependent changes. The cyanobacterium *Limnospira platensis* (formerly *Arthrospira platensis*) exhibited a reduction in protein content in response to both ZnONPs (<100 nm) and TiO_2_NPs (21 nm), with the effect being proportional to nanoparticle concentration [34]. The microalga *Nannochloropsis oculata* (Ochrophyta) maintained protein levels at low ZnONPs (size 10–30 nm) concentrations, but these declined at higher doses [9]. In *Chlorella sorokiniana* (Chlorophyta), protein content increased by about 2-fold at TiO_2_NP (size 10–50 nm) doses ≥ 0.079 mg/L, possibly reflecting a detoxification or adaptation mechanism [8]. In *Isochrysis galbana* (Haptophyta), exposure to TiO_2_ nanoparticles up to 50 mg/L decreased protein content, while in *Chaetoceros muelleri* (Ochrophyta), a reduction occurred at concentrations up to 400 mg/L [10,36]. Similarly, in *Dunaliella tertiolecta* (Chlorophyta), protein reduction was observed mainly at high TiO_2_NPs (size 25 nm) concentrations, whereas low doses (0.01–10 mg/L) had no effect [37]. For *Chlorella* sp. (Chlorophyta), exposure to 50 mg/L ZnONPs (size < 100 nm) increased both biomass and protein content 1.68-fold compared to the control on day 2 of incubation. In contrast, higher concentrations (100–200 mg/L) significantly reduced both parameters, though growth was not fully inhibited [38]. Another study reported that *Nannochloropsis oculata* (Ochrophyta) biomass increased proportionally with ZnONPs (size 10–30 nm) concentration, an effect interpreted as an adaptive stress response [9].

A relevant indicator of the adaptation of photosynthetic microorganisms to stress factors is the change in carbohydrate content. An increase in carbohydrate levels reflects functional adaptability, indicating the maintenance or even optimization of metabolic processes under adverse conditions. In our study, the application of low concentrations of ZnONPs stimulated carbohydrate synthesis in the cyanobacterium *Nostoc linckia*, while high concentrations inhibited this process. In the case of TiO_2_NPs, an increase in carbohydrate content was observed at medium and high concentrations.

Studies on various microalgae and cyanobacteria have shown that nanoparticles can induce significant changes in carbohydrate content. In *Limnospira platensis*, high concentrations of ZnONPs (<100 nm) caused a marked increase in carbohydrates, whereas exposure to TiO_2_NPs (size 21 nm) produced a relatively constant result regardless of concentration [34]. Low concentrations of CuONPs (size 50 nm) stimulated carbohydrate accumulation in the biomass of *Nostoc linckia* [26].

In *Chlorella sorokiniana* (Chlorophyta), carbohydrate content doubled at the highest concentration of TiO_2_ nanoparticles with size of 10–50 nm, indicating a physiological stress response [8]. In contrast, *Chaetoceros muelleri* (Ochrophyta), exposed to 100–400 mg/L TiO_2_NPs exhibited a slight decrease in carbohydrate level [36]. Similarly, in *Isochrysis galbana* (Haptophyta), exposure to 50 mg/L TiO_2_NPs resulted in a 42.8% reduction in carbohydrates, which correlated with decreased protein content [10]. A different pattern was observed in *Dunaliella tertiolecta* (Chlorophyta), where low TiO_2_NPs (size 25 nm) concentrations (0.01–10 mg/L) did not significantly alter carbohydrate content [37].

Altogether, these results indicate that carbohydrate accumulation may reflect an adaptive cellular response to stress, whereas reductions, especially at high nanoparticle concentrations, suggest that the cells’ adaptive capacity has been exceeded, leading to destructive effects.

Studies on various microalgae and cyanobacteria have revealed significant changes in photosynthetic pigments under the action of metallic nanoparticles. In *Nostoc linckia*, exposure to CuONPs (size 50 nm) at concentrations similar to those applied in the present study increased chlorophyll and carotenoid content [26]. In *Limnospira platensis*, low concentrations of ZnONPs did not significantly affect pigment levels, but higher doses caused reductions of more than 50% in chlorophyll and over 40% in carotenoids. In contrast, TiO_2_NPs did not significantly alter these parameters at most tested concentrations, except at 20 mg/L, highlighting the nanoparticle type-dependent nature of the response [34].

The mechanisms of ZnONPs toxicity on photosynthetic systems are attributed to several factors. For instance, in *Chlorella* sp. (Chlorophyta) exposed for 96 h to ZnONPs of different sizes (30, 90, and 200 nm) and to Zn^2+^ ions, the most substantial reduction in chlorophyll occurred in the presence of ions, followed by the effect of nanoparticles. This decrease was explained by the aggregation and adhesion of ZnONPs on the cell surface, which limited light availability and its utilization [39]. Similar observations were reported for *Chlorella* sp. exposed to 50 mg/L ZnONPs (size < 100 nm), where on day 2, chlorophyll a increased 1.35-fold, chlorophyll b 1.28-fold, and carotenoids 1.56-fold, followed by declines below control levels at higher concentrations or with prolonged exposure [38].

The susceptibility of different microalgal species to TiO_2_NPs and ZnONPs toxicity is well documented. In *Tetraselmis suecica* (Chlorophyta) a 50% reduction in chlorophyll a and >50% in carotenoids was observed at both 7.9 mg/L and 400 mg/L TiO_2_NPs (size 10–70 nm) [8,40]. *Chaetoceros muelleri* (Ochrophyta), exposed for 10 days to high TiO_2_NPs concentrations, exhibited a drastic reduction in chlorophyll [36]. In *Chlorella vulgaris* (Chlorophyta), ZnONPs with size < 100 nm caused significant decreases in chlorophyll a after 24 h, in a concentration-dependent manner associated with growth inhibition, whereas *Pseudokirchneriella subcapitata* lost substantial chlorophyll a after 72 h of exposure [39,41]. At higher concentrations ZnONPs (100–500 mg/L) the decline in chlorophyll a in *Chlorella vulgaris* was attributed to light shading by nanoparticle aggregates or oxidative stress induced photosystem damage. Carotenoids followed a similar downward trend, indicating impairment of the entire photosynthetic apparatus and reduced photo-protective antioxidant capacity [38]. These results show that metal oxide nanoparticles significantly influence pigment metabolism of microalgae and cyanobacteria, with the magnitude and direction of effects depending on the nanoparticle type, applied concentration, and species-specific characteristics.

In addition to primary photosynthetic pigments (chlorophyll and carotenoids), photosynthetic organisms, including cyanobacteria and some marine microalgae, also contain accessory pigments such as phycobiliproteins, which extend the light absorption spectrum and optimize energy transfer to the photosynthetic reaction centers. In the cyanobacterium *Nostoc linckia*, phycobiliproteins play an essential role in solar energy capture and transfer. However, their levels can decline to undetectable values under exposure to xenobiotics, including nanoparticles [20,26,34].

Previous studies demonstrated that CuONPs induced a concentration-dependent decline in phycobiliproteins in *Nostoc linckia*. Among the three phycobilin pigments, phycocyanin, allophycocyanin, and phycoerythrin, only phycoerythrin remained stable, while the others decreased. This reduction was accompanied by an increase in chlorophyll and carotenoid content [26]. In contrast, the present study revealed that ZnONPs and TiO_2_NPs caused a decrease in phycobiliproteins concurrently with a reduction in chlorophyll in *Nostoc linckia*, with phycoerythrin again remaining the most stable pigment component.

Photoactive nanoparticles such as ZnONPs and TiO_2_NPs generate reactive oxygen species (ROS) upon light absorption. These ROS preferentially target photosynthetic structures in close proximity, particularly the membranes housing phycobilisome pigment. The internal components of the phycobilisome, phycocyanin and allophycocyanin, located near photosystem II, are directly exposed to oxidative stress, resulting in accelerated degradation. In contrast, phycoerythrin, positioned on the external side of the phycobilisome, is less exposed to ROS and structurally protected, which explains why its content remains relatively constant under nanoparticle-induced stress.

Studies on the effects of nanoparticles on cyanobacteria and microalgae have demonstrated that lipid metabolism and malondialdehyde MDA levels are key indicators of cellular adaptation to oxidative stress. Membrane lipids are essential for maintaining cell integrity and functionality, and alterations in their composition can reflect both adaptive responses and damage processes. MDA, the main product of lipid peroxidation, serves as a reliable marker of oxidative damage: low levels suggest effective protection, whereas elevated levels indicate structural impairment of membranes.

Metal oxide nanoparticles such as ZnONPs and TiO_2_NPs are studied not only for their effects on cellular metabolism but also as potential tools to stimulate lipid synthesis for biofuel production. This effect is attributed to moderate oxidative stress, which redirects metabolism towards lipid accumulation [33]. Studies have shown that *Nostoc linckia* responded to ZnONPs and TiO_2_NPs with a significant increase in lipid content, whereas CuONPs induced this effect only at high concentrations [26]. Similar results have been reported in other species. *Isochrysis galbana* COR-A3 exhibited lipid reduction at 50 mg/L TiO_2_NPs after 96 h [10], while *Chaetoceros muelleri* lost >40% of lipids at 400 mg/L TiO_2_NPs [36]. In *Limnospira platensis*, high concentrations of ZnONPs reduced lipids, whereas equivalent doses of TiO_2_NPs stimulated lipid accumulation [34]. In *Chlorella vulgaris*, ZnONPs (size < 100 nm) increased lipid content at moderate concentrations (10–50 mg/L), interpreted as an adaptive response, but higher doses caused lipid decreases, indicating severe toxicity [38].

The determination of MDA levels completed the assessment of oxidative stress intensity. In *Nostoc linckia*, exposure to ZnONPs and TiO_2_NPs stimulated lipid accumulation while maintaining low MDA levels, suggesting controlled oxidative stress rather than membrane damage. In contrast, high CuONPs concentrations increased MDA, reflecting intense oxidative stress, whereas low doses stimulated lipid accumulation without raising MDA, indicative of an adaptive response [26]. In *Limnospira platensis*, ZnONPs (<100 nm) reduced MDA, likely by limiting biosynthetic processes, while TiO_2_NPs (size 21 nm) decreased MDA at low concentrations and maintained control levels at higher doses [34]. Data from other studies support that metallic nanoparticle frequently induce oxidative stress, as evidenced by elevated MDA levels. For example, in *Chlamydomonas reinhardtii*, exposure to up to 50 mg/L TiO_2_NPs (size 21 nm) significantly increased MDA, which correlated with a reduction in lipid content [42].

In this study we obtained an inverse relationship between lipid content and MDA levels in Nostoc linckia. Both ZnONPs and TiO_2_NPs stimulated lipid accumulation across all concentrations tested, while at the same time MDA levels were significantly reduced compared to the control. This suggests that lipid accumulation occurred under conditions of controlled oxidative stress, without evidence of membrane damage. In other words, the increase in lipid content appears to reflect an adaptive metabolic response rather than a consequence of enhanced lipid peroxidation.

The results of this study show that *Nostoc linckia* displayed a high level of tolerance to both ZnO and TiO_2_ nanoparticles, as evidenced by biomass stimulation and the maintenance of low oxidative stress markers. This apparent resistance is likely multifactorial. On the one hand, the ecological plasticity of Nostoc may provide an inherent capacity to adapt to changing stressors. On the other hand, the bioavailability of the tested nanoparticles differs markedly: TiO_2_NPs are largely insoluble under culture conditions and exert their effects mainly through surface interactions and photocatalytic ROS generation [7], while ZnONPs are known to gradually release Zn^2+^ ions in amounts that often determine their toxicity [43,44,45]. The small quantity of EDTA in the medium, introduced solely to stabilize micronutrients, might have slightly influenced Zn^2+^ speciation but is unlikely to have played a major role. Taken together, these factors suggest that the stimulatory effects observed here are consistent with a hormetic-type adaptive response, rather than the absence of stress.

## 5. Conclusions

Exposure of *Nostoc linckia* cultures to ZnO and TiO_2_ nanoparticles resulted in significant modifications of biomass, biochemical composition, and photosynthetic pigments. Biomass accumulation was stimulated by both types of nanoparticles, although the response was concentration-dependent. TiO_2_NPs consistently enhanced growth at all tested concentrations, with the highest increase of 41% recorded at 30 mg/L. In contrast, ZnONPs promoted biomass accumulation primarily at low and medium concentrations (0.1–20 mg/L), suggesting a hormesis-type effect, while the highest concentration produced no significant change.

At the biochemical level, protein content remained largely stable, with only moderate decreases observed at 20 mg/L ZnONPs and 30 mg/L TiO_2_NPs. Carbohydrate levels were more variable, showing a significant increase at 1 mg/L ZnONPs and decreases at higher concentrations, while changes induced by TiO_2_NPs were not significant. Lipid content increased across all treatments, reaching maximum values at 1 mg/L ZnONPs, whereas MDA levels were consistently reduced compared to the control. These findings indicate that nanoparticle concentration is a significant factor influencing both lipid accumulation and oxidative stress.

Photosynthetic pigments also showed distinct responses. ZnONPs stimulated chlorophyll and carotenoid synthesis at low to moderate concentrations, while TiO_2_NPs caused moderate reductions in carotenoids. Regarding phycocyanin, allophycocyanin, and phycoerythrin, most treatments produced no significant changes relative to the control, except for strong reductions in phycocyanin at 10–30 mg/L ZnONPs. While individual pigments showed limited variability, the total phycobiliprotein content displayed a clear and significant decrease at high concentrations of both nanoparticle types.

Taken together, these results demonstrate the metabolic robustness of *Nostoc linckia* and its capacity to adapt to stress induced by photoactive nanoparticles. The simultaneous stimulation of biomass growth and lipid accumulation, along with reduced MDA levels, reflects a regulated metabolic adjustment toward the synthesis of valuable metabolites. This adaptive response highlights the biotechnological potential of *Nostoc linckia*, particularly in processes where controlled nanoparticle exposure could be applied to enhance the production of metabolites of interest. Nevertheless, these conclusions should be considered in the context of the experimental scale, and further studies will be useful to confirm and broaden the applicability of the present findings.

## Data Availability

The original contributions presented in this study are included in the article. Further inquiries can be directed to the corresponding author.
